# Editorial: Flexible Biosensors and Intelligent Medical Devices in Health and Disease

**DOI:** 10.3389/fbioe.2022.849617

**Published:** 2022-06-02

**Authors:** Jian Yang

**Affiliations:** Faculty of Intelligent Manufacturing, Wuyi University, Jiangmen, China

**Keywords:** flexible biosensors, medical devices, machine learning, artificial intelligence, health care

Recent advances have been made in biosensors and medical devices for diagnosis and health monitoring of various diseases. Flexible biosensors accelerate the development of wearable biomedical technologies and intelligent medical devices help doctors or patients to understand clinical characteristics and biomedical data. This Research Topic focuses on both the hardware and software of intelligent biomedical devices, including flexible or wearable biosensors, machine learning algorithms, and artificial intelligence-based technologies in health and disease. Flexible or wearable biosensors provide a new efficient way to monitor and obtain medical data, on the other hand, artificial intelligence shows its effectiveness in massive medical data mining and clinical diagnosis. With the recent development of flexible electronics and artificial intelligence, both innovative machine learning algorithms and high-performance data processing, and intelligent medical devices are applied to meet the need of personalized medicine and patient healthcare in various areas.

In this collection, a wearable ear device system was proposed by Lian et al. to address facial emotion recognition disorders with a deep-learning based multi-model physiological signal recognition algorithm. Multiple data from sensors such as heart rate, triaxial acceleration, skin electricity, body temperature, and facial images were analyzed and proved the effectiveness of this system for patients with potential nervous system diseases. As for post-surgical patients, especially those with upper extremity lymphedema, a portable monitoring and auxiliary treatment device was presented by Yanmin et al. to help them better rehabilitate. The pressure sensing layer embedded in the device can constantly measure and compare the change of arm circumference in different parts of the upper limb, which can provide the patients with early detection and effective control. Gori et al. exhaustively reviewed the biomedical and tissue engineering strategies to control foreign body reactions to invasive neural electrodes in recent years. From molecular mechanism and cellular components of foreign body reactions to design and geometry of the electrodes, they mentioned the development of innovative and advanced functional biomaterials including MEMS polymer materials and hydrogels and the interface-microenvironment interaction affected by anti-inflammatory or anti-fibrotic drugs, intraneural interfaces, or neuroprostheses that are beneficial for amputees to restore their sensorimotor limb function. The authors also indicated that next-generation intraneural electrodes produced by current microfluidic, microscale, and nanoscale technologies deserved further investigation. Flexible or wearable biosensors have promoted these advances in disease diagnosis and the healthcare of various patients.

On the other side, it is a burden for doctors or patients to treat and analyze huge medical data, especially image data. Computational intelligent algorithms such as machine learning, deep learning, and transfer learning have been applied to medical imaging classification, showing predictable advantages in massive data treatment and clinical diagnosis. For instance, Li et al. compared eight classical supervised machine learning methods, two deep learning models, and one transfer learning model with more than 1,000 radiological samples. The results showed that transfer learning is most effective in the classification of colorectal cancer lymph node metastasis with a better accuracy; they did not need to train the model and it could be applied to a smaller medical image dataset. As for childhood cataract patients, Lin et al. developed an intelligent system based on machine learning models and the features of optical coherence tomography images, 200 eye data of 132 patients in 8 years were collected and analyzed, demonstrating that the best corrected visual acuity of childhood cataract patients in 3 and 5 years can be predicted with a small error. Similarly, Huang et al. combined different machine learning algorithms with the radiomics features of multiparameter MRI and clinicopathological characteristics to predict the tumor shrinkage pattern of breast cancer patients prior to neoadjuvant chemotherapy. A total of 4,198 features in segmented MRI sequences and a set of clinicopathological data including age, menstrual state, biomarkers, and receptors for each patient were treated and analyzed. The multilayer perception neural network achieved a higher area under the curve and better accuracy, it established a feasibility for early diagnosis and prediction of breast cancer. Because of the increasing incidence rate of breast cancer, Dou et al. investigated kernel function selection and combined a genetic algorithm, particle swarm optimization, and simulated annealing with support vector machine algorithm. The optimization algorithm could significantly improve the diagnosis efficiency of their medical institution.

Magnetic resonance images play an important role in clinical disease diagnosis and physiological mechanism. However, the automatic segmentation of the left ventricle wall in the four-chamber view of MRI is still a challenge. Zhang et al. proposed a dense recurrent neural network algorithm to accurately achieve left ventricle wall segmentation in a four-chamber view of MRI sequences, this method provided compensation for the first long short-term memory cells and made the hidden information in the cells more visible, with better cardiac state estimation. Similarly, Duan et al. proposed a multi-branch feature fusion network to achieve the real-time segmentation of liver lesion images for colonoscopy, which can detect rectal polyps with high accuracy and read-time performance. Deng et al. designed medical ultrasound remote control software which can use mobile devices to remotely control ultrasound devices in the same local area network, Yanbo et al. utilized a fuzzy neural network control system model to improve the removal efficiency of toxic and refractory organic water in waste water.

Therefore, it is clearly seen that artificial intelligence-based technologies have been widely studied in various areas of disease diagnosis, which are especially helpful for computer-aided diagnosis. This topic showed recent advances in *Flexible Biosensors and Intelligent Medical Devices in Health and Disease*.

